# Effects of Lipoic Acid Supplementation on Activities of Cyclooxygenases and Levels of Prostaglandins E_2_ and F_2_ *α* Metabolites, in the Offspring of Rats with Streptozotocin-Induced Diabetes

**DOI:** 10.1155/2016/9354937

**Published:** 2016-11-30

**Authors:** Hisham Y. Al-Matubsi, Ghaleb A. Oriquat, Mahmoud Abu-Samak, Othman A. Al Hanbali, Maher D. Salim

**Affiliations:** ^1^Faculty of Pharmacy, University of Petra, Amman, Jordan; ^2^Faculty of Pharmacy and Medical Sciences, Al-Ahliyya Amman University, Amman, Jordan; ^3^Faculty of Pharmacy, Applied Science University, Amman, Jordan; ^4^Faculty of Pharmacy, Al-Zaytoonah University, Amman, Jordan; ^5^Middle East University, Amman, Jordan

## Abstract

*Background.* Our aim was to evaluate the protective effect of lipoic acid (LA) on fetal outcome of diabetic mothers.* Methods.* Diabetes was induced in female rats using streptozotocin and rats were made pregnant. Pregnant control (group 1; *n* = 9; and group 2; *n* = 7) or pregnant diabetic (group 3; *n* = 10; and group 4; *n* = 8) rats were treated daily with either LA (groups 2 and 4) or vehicle (groups 1 and 3) between gestational days 0 and 15. On day 15 of gestation, the fetuses, placentas, and membranes were dissected, examined morphologically, and then homogenized, to measure cyclooxygenase (COX) activities and metabolisms of prostaglandin (PG) E_2_ (PGEM) and PGF_2_ *α* (PGFM) levels. The level of total glutathione was measured in the maternal liver and plasma and in all fetuses.* Results.* Supplementation of diabetic rats with LA was found to significantly (*p* < 0.05) reduce resorption rates in diabetic rats and led to a significant (*p* < 0.05) increase in liver, plasma, and fetuses total glutathione from LA-TD rats as compared to those from V-TD. Decreased levels of PGEM and elevated levels of PGFM in the fetuses, placentas, and membranes were characteristic of experimental diabetic gestation associated with malformation. The levels of PGEM in malformed fetuses from LA-TD mothers was significantly (*p* < 0.05) higher than those in malformed fetuses from V-TD rats.* Conclusions.* LA treatment did not completely prevent the occurrence of malformations. Thus, other factors may be involved in the pathogenesis of the diabetes-induced congenital malformations.

## 1. Background

Mothers suffering from diabetes mellitus have high chances of loss of pregnancy and giving birth to babies with congenital malformations. These congenital malformations most commonly involve the central nervous, cardiovascular, and skeletal systems [1]. There has been an ongoing research that seeks to identify the factors that have been involved in diabetic embryopathy, including increased levels of glucose and ketone bodies [2, 3] and reduced levels of arachidonic acid and prostaglandin E_2_ (PGE_2_) [4, 5]. Furthermore, several studies suggested that disequilibrium of oxidant-antioxidants levels prevalent in diabetic patients is a potential cause of these aberrations [6–11]. Increased formation of reactive oxygen species in association with increased teratogenesis has been demonstrated in rat embryos cultured* in vitro* in medium containing high levels of glucose [7]. The addition of antioxidants and superoxide dismutase to the medium decreased the rate of embryonic dysmorphogenesis, which suggests the involvement of reactive oxygen species in the teratogenic process [8].* In vivo* studies using several models of diabetes suggested that administration of vitamin E [9], vitamin C [10], and lipoic acid [11] was found to be useful in improving fetal outcome in* in vivo* models of diabetic embryopathy.

Lipoic acid (LA, also known as alpha-lipoic acid or thioctic acid) is found inside every cell of the body. Lipoic acid is a potent antioxidant, capable of inducing its activity in both a lipophilic and an aqueous milieu [12, 13]. It also helps to regenerate both fat and water soluble antioxidant vitamins [14] and improve sugar metabolism and energy production [15, 16]. Furthermore, *α*-LA was shown to reverse apoptosis, ameliorate mitochondrial deformation, and increase the amount of mitochondrial DNA [17].

A healthy body makes enough LA to supply its energy requirements; therefore, there is no daily requirement for this supplement. However, several medical conditions appear to be accompanied by low levels of LA—specifically, diabetes, liver cirrhosis, and heart disease [14]—which suggests that supplementation would be helpful.

At present, the exact mode of action of LA has not been fully elucidated. This research study is designed to investigate the protective nature of LA on fetal outcome while determining the processes involved in diabetic fetopathy. To achieve this, the study will examine the effects of LA on the activity of cyclooxygenases and the levels of prostaglandins E_2_ and F_2_ *α* metabolites in diabetic rats.

This study will make major contribution to our nation as it will reduce the rate of congenital malformation and will be of considerable benefit to health sector since congenital malformation imposes a substantial cost to the health budget and emotional burdens upon society.

## 2. Methods

### 2.1. Experimental Animals

All animal studies were reviewed and approved by the Animal Ethics Committee of University of Petra (no. ANIM: LAGES-2014-04). The guide for the care and use of laboratory animals was followed.

Nulliparous female rats from a local Wistar-derived strain, 60 to 70 days of age with initial weights of 220 ± 30 g, were used. They were housed in rooms maintained at a temperature of 21°C ± 3°C and 12 : 12 h light/dark cycle. A commercial diet and tap water were provided* ad libitum*.

### 2.2. Experimental Diabetes Induction

The rats were randomly distributed to four groups, two control groups (pregnant nondiabetic; groups 1 and 2) and two diabetic groups (groups 3 and 4). Diabetes was induced in female rats from groups 3 (*n* = 10) and 4 (*n* = 8) by a single intraperitoneal (IP) injection of freshly prepared streptozotocin (STZ; Sigma-Aldrich Chemical Co., Poole, UK; 65 mg/kg, dissolved in citrate buffer, 100 mmol/L; pH 4.5), while control animals groups 1 (*n* = 9) and 2 (*n *= 7) were injected with a buffer solution (an equivalent volume of STZ injection). One week after the injections, venous tail blood was collected and hyperglycemia was confirmed with blood glucose levels greater than 11 mmol/L using a glucometer.

### 2.3. Mating

The rats (both controls and diabetic) were mated with healthy nondiabetic males overnight. The presence of a sperm mucus plug in the vagina the following morning signified pregnancy. Further, the presence of spermatozoa was designated as day zero of pregnancy. Pregnancy was confirmed by subsequent increases in body weight.

### 2.4. Lipoic Acid Induction

Thereafter, rats were daily injected IP with either LA (30 mg/kg body weight in Tris buffer with pH 7.4; groups 2 and 4) or vehicle only (groups 1 and 3) between gestational days 0 and 15.

### 2.5. Sample Collection

At day 15 of gestation, pregnant diabetic and control rats were sacrificed by cervical dislocation, and maternal blood samples were collected by cardiac puncture. In each sacrificed rat, the uterus was exposed by cesarean section, and the number of implantation and resorption processes was recorded. The live fetuses, along with their placentas and membranes, were dissected out of the uterine horns, rinsed carefully in phosphate buffered saline (PBS), and carefully labeled. Overall growth and differentiation of the fetuses were quantified by direct measurement of crown-to-rump length (CRL). The fetuses were examined for their general morphology, and the presence of any disturbed fetal development, such as an open neural tube or growth retardation, and the appearance of specific parts and organs (head, ear, legs, tail, and body rotation) were also noted. This examination was conducted by a researcher masked to the various experimental groups. Fetuses with apparent anomalies in one or more of these structures were considered “malformed.” Fetal, placental, and membrane weights were also recorded. The fetuses, placentas, and membranes were then snap-frozen in liquid nitrogen and kept at −80°C for subsequent analysis.

### 2.6. Sample Preparation and Assays

To rule out the possible presence of COX-1 inhibitor in association with pregnancy, we examined the activity of COX-1* in vitro *by the addition of supernatants from the homogenates of placentas, which could have contained such an inhibitor, to the incubated media, and found that such activity was not affected.

The COX activity assays were determined according to procedures used previously [18]. Protein was determined using a modification of Bradford's method [19]. The specific activity of each enzyme was determined by dividing its activity by the protein concentration in the sample (U/mg protein). The intra- and interassay coefficients of variation for COX were 2.6% and 5.4%, respectively.

Circulating prostaglandin (PG) PGF_2_ *α* and PGE_2_ are rapidly converted* in vivo* to their 13,14-dihydro-15-keto metabolites PGFM and PGEM, respectively, [20, 21] with more than 90% cleared from circulation by a single passage through the lungs. Although levels of the enzymes that metabolize prostaglandins vary in different tissues, it is considered that measurement of their metabolites is used to provide a reliable estimate of actual PGF_2_ *α* and PGE_2_.

PGFM and PGEM were measured by enzyme immunoassays (EIA) [20] with a sensitivity of 11 pg/mL and 120 pg/mL, respectively. The intra- and interassay coefficients of variation for PGFM were 10% and 21% and for PGEM were 8% and 8%, respectively.

Biochemical parameters were determined for all individual fetuses and placentas, while membranes from each group were pooled to allow determination of all parameters in the same sample. PGs metabolites were also assayed in maternal blood.

Liver samples from the maternal rats were removed, then snap-frozen in liquid nitrogen, and kept at −80°C for subsequent analysis. Liver and plasma total glutathione levels, a sensitive indicator of oxidative stress, were determined and expressed as *μ*mol/g tissue and *μ*mol, respectively. The level of total glutathione was measured also in the fetuses of all groups and expressed as *μ*mol/mg of protein. The kits for COX, EIA for both PGFM and PGEM, and total glutathione levels were from the Cayman Chemical Company (Ann Arbor, MI, USA).

### 2.7. Statistical Analysis

Statistical analysis of the present study data was performed by a biostatistician using Shapiro-Wilk, Kruskal-Wallis, Tukey, and/or Mann-Whitney one-way analysis of variance rank sum tests (SPSS statistical software; SPSS Inc., Chicago, IL, USA). Statistical difference for overall malformation between both diabetic groups (vehicle-treated diabetic and LA-treated diabetic) was calculated using a chi-squared test. Results were considered statistically significant when the* p *value was smaller than 0.05. Data are presented as means ± SEM.

## 3. Results

### 3.1. Glucose and Body Weight

The average glucose levels in random blood samples from diabetic pregnant rats were significantly higher (*p* < 0.05) (five times) than comparable samples obtained from both groups of control pregnant rats (Table 1).

Furthermore, the average glucose levels in samples obtained from LA-treated diabetic (LA-TD) rats were also significantly higher (*p* < 0.05) than samples obtained from both groups of control rats. Daily injections of LA did not normalize blood glucose levels of diabetic rats to the control rats and did not significantly affect the blood sugar concentrations of both diabetic and control rats.

As shown in Table 1, supplementation of control rats with LA (30 mg/kg) for 15 days did not have any effect on body weight gain. However, supplementation of diabetic rats with LA was found to improve body weight gain compared to V-TD group but did not normalize weight gain to both groups of control rats.

### 3.2. Gross Morphological Study

The mean number of implantation surgeries per litter was similar in all studied groups (Table 1).

In diabetic rats, LA supplementation significantly reduced (*p* < 0.05) resorption rates from 5.80 ± 2.15 in V-TD rats to zero in LA-TD rats.

Fetal body mean weight in V-TD rats was significantly decreased (*p* < 0.05) in comparison to both groups of control and LA-TD rats.

Placental mean weight from both groups of diabetic rats was significantly higher (*p* < 0.05) than those in V-TC rats. In contrast, membrane mean weight in both groups of control rats was significantly higher (*p* < 0.05) than those in both diabetic rat groups.

**Figure 1:**
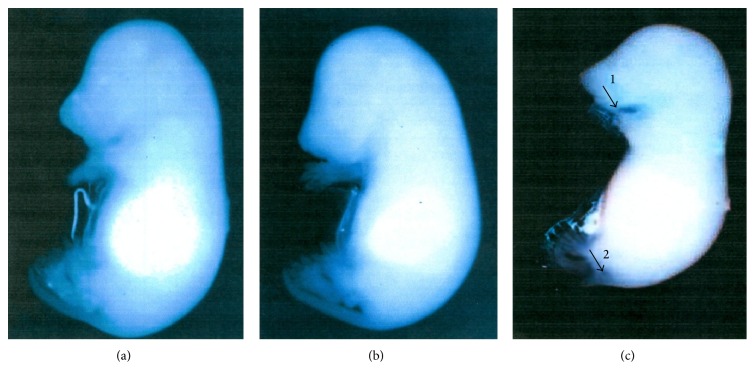
(a) Control fetus, (b) nonmalformed fetus of diabetic rat, and (c) malformed fetus of diabetic rat. Arrows indicate the position of malformation (1: short mandible, 2: short tail).

While no gross morphological malformations were detected in the fetuses of the both groups of control mothers, experimentally induced diabetes was associated with the presence of malformations (Figure 1).

To estimate embryonic development, we measured fetal crown-rump length (CRL, Table 2). In offspring of V-TD rats, a significant decrease (*p* < 0.05) in mean CRL was observed, in comparison with offspring of V- and LA-TC or LA-TD rats. Supplementation of diabetic rats with LA increased CRL in their offspring and was able to normalize the measure to those in V- and LA-TC rats.

Administration of LA to diabetic rats for 15 days during gestation marginally reduced the occurrence of malformations (Table 3). The incidence of malformation in V-TD reached approximately 67% (32 of 48 litters) as compared to 42% in LA-TD (25 of 59 litters). Thus, the overall malformation variance between both diabetic groups did not reach statistical significance (*p* = 0.06).

In the V-TD rats, 41.66% of fetuses had growth retardation, as represented by CRL, 39.58% had short mandibles, 31.25% had open neural tubes, 12.5% had malrotations, and 2.08% had short tails. However, in LA-TD rats, the corresponding proportions were 15.25%, 10.17%, 13.56%, 5.09%, and 0.0%, respectively. Some fetuses exhibited a single malformation, while others had multiple anomalies.

### 3.3. Total Glutathione Levels

To assess the ability of LA to reduce the maternal oxidative stress associated with diabetes, we measured the level of total glutathione in the maternal liver and plasma.

As seen in Table 4, maternal liver of the V-TD rats was associated with significantly reduced (*p* < 0.05) glutathione content in comparison with those in V- and LA-TC rats.

Supplementation of diabetic rats with LA leads to a significant increase (*p* < 0.05) in liver total glutathione in comparison with V-TD rats, although such supplementation failed to normalize glutathione content to those in the liver of both groups of control rats. Furthermore, LA treatment significantly increased (*p* < 0.05) the content of total glutathione in the plasma of diabetic rats compared with those in V-TD rats. This value was not found to be significantly different from that observed in both groups of control rats.

To find out whether maternal diabetes decreases fetal total glutathione levels and if the LA supplementation reverses fetal total glutathione levels, we measured the level of total glutathione in fetuses of all groups (both control and diabetic).

The total glutathione contents in fetuses of V- and LA-TD rats were 51.17% and 45.17% less than in those of V- and LA-TC rats, respectively. On the other hand, the contents of total glutathione in fetuses of both diabetic rats were significantly lower (*p* < 0.05) than those in both control groups.

LA supplementation to diabetic rats significantly increased (*p* < 0.05) total glutathione content of their fetuses as compared with fetuses from V-TD rats.

### 3.4. Cyclooxygenase Activity in Fetuses

The activity of COX-1 in the fetuses, placentas, and membranes from all control and diabetic mothers represented a small fraction of total COX activity compared with that of COX-2. The presence of a COX-1 inhibitor in the V-TC and V-TD rats was investigated and found to be negative.

The activity of COX-1 showed no significant variations among fetuses from all groups (Table 5). However, the activities of COX-2 in malformed fetuses from V-TD and LA-TD were significantly lower (*p* < 0.05) compared with fetuses from V-TC, LA-TC, nonmalformed V-TD, and nonmalformed LA-TD mothers. Statistically, there was no significant difference between both control and nonmalformed diabetic fetuses.

### 3.5. Cyclooxygenase Activity in Placentas

There was a significant decrease in the activities of both COX isoenzymes in the placentas of malformed fetuses from both diabetic pregnancies. A significant decrease (*p* < 0.05) of COX-1 activity was associated with placentas from malformed V-TD and LA-TD fetuses compared with placentas of nonmalformed fetuses (Table 6).

A similar trend was found in activities of COX-2; the activities of COX-2 in V-TD and LA-TD placentas from malformed fetuses were significantly lower (*p* < 0.05) than nonmalformed fetuses.

The activities of COX-2 in the placentas from both control fetuses were significantly higher (*p* < 0.05) compared with those from both nonmalformed and malformed fetuses of diabetic mothers.

Supplementation of diabetic rats with LA leads to a significant increase (*p* < 0.05) in the activities of COX-2 in the placentas from malformed fetuses in comparison with those from V-TD rats. Thus, LA treatment to diabetic mothers failed to normalize activities of COX-2 to the control rats.

### 3.6. Cyclooxygenase Activity in Membranes

The total activity of COX in membranes from the both diabetic was significantly lower (*p* < 0.05) in comparison to membranes from both the control fetuses (Table 7). Furthermore, the activity of total COX in membranes from nonmalformed fetuses of LA-TD was significantly higher (*p* < 0.05) compared with the nonmalformed fetuses of V-TD mothers. The activities of COX-1 in membranes showed no significant variations among fetuses from all groups.

The activities of COX-2 in membranes of malformed fetuses from V-TD and LA-TD were significantly lower (*p* < 0.05) than those in the membranes of fetuses from V-TC, LA-TC, and nonmalformed fetuses from V-TC and LA-TD mothers. Furthermore, the activity of COX-2 in membranes from nonmalformed fetuses of LA-TD was significantly higher (*p* < 0.05) than those in nonmalformed fetuses from V-TD rats.

### 3.7. Levels of PGEM and PGFM in Fetuses

Decreased levels of PGEM and elevated levels of PGFM in the fetuses, placentas, and membranes were characteristic of both experimental diabetic gestation groups associated with malformation.

As shown in Table 8, the mean levels of PGEM in fetuses (nonmalformed and malformed) from both groups of diabetic mothers were significantly lower (*p* < 0.05) than those in both control groups.

The mean value for PGEM in nonmalformed fetuses was significantly higher (*p* < 0.05) than in the malformed fetuses from V-TD and LA-TD mothers, although the levels of PGEM in malformed fetuses from LA-TD mothers were significantly higher (*p* < 0.05) than those in malformed fetuses from V-TD rats. LA treatment to diabetic mothers failed to normalize levels of PGEM to that of both groups of control rats.

In contrast to PGEM, the greatest increases in PGFM were found in fetuses from malformed V-TD and LA-TD mothers and were significantly higher (*p* < 0.05) than those in nonmalformed V-TD and LA-TD mothers.

The mean concentrations in malformed fetuses of both diabetic groups were also significantly higher (*p* < 0.05) than that in V-TC and LA-TC.

### 3.8. Levels of PGEM and PGFM in Placentas

In placentas from both groups of diabetic fetuses, PGEM levels were significantly lower (*p* < 0.05) than the mean values in control groups.

The decrease in placenta PGEM associated with malformed fetuses was even more precipitous in both V-TD and LA-TD and below than that in the control groups and those of nonmalformed samples of both diabetic fetuses (*p* < 0.05).

PGFM was also raised in placentas of both diabetic groups in a pattern similar to that in fetuses from both groups of diabetic mothers (Table 9). The increase in placentas from malformed V-TD and LA-TD fetuses was significantly greater (*p* < 0.05) than those from nonmalformed fetuses of both diabetic and control groups. The average levels in placentas from nonmalformed V-TD and LA-TD fetuses were significantly higher (*p* < 0.05) than in V-TC and LA-TC fetuses.

Although the average levels in placentas from malformed (V-TD and LA-TD) fetuses were higher than those from placentas without malformation, this difference was not statistically significant.

### 3.9. Levels of PGEM and PGFM in Membranes

PGEM levels in the membranes of both diabetic fetuses were significantly lower (*p* < 0.05) than those in the controls.

In the membranes of nonmalformed V-TD and LA-TD fetuses, the mean levels of PGEM were significantly lower (*p* < 0.05) than those in the V-TC and LA-TC.

The mean levels of PGEM in the membranes of malformed V-TD and LA-TD fetuses were significantly below (*p* < 0.05) those of nonmalformed fetuses of both diabetic groups. Further, supplementation of diabetic mothers with LA significantly increased (*p* < 0.05) levels of PGEM in membranes of fetuses (nonmalformed and malformed) compared with those in the V-TD fetuses.

The smallest increases in PGFM were observed in fetal membranes (Table 10). The levels of PGFM in membranes of fetuses from both diabetic groups were significantly higher (*p* < 0.05) than those in the control groups.

The mean PGFM levels in membranes from V-TD nonmalformed and malformed fetuses were significantly higher (*p* < 0.05) than those in the V-TC and LA-TC.

In a pattern similar to that found in membranes from V-TD fetuses, mean PGFM levels in membranes from LA-TD nonmalformed and malformed fetuses were significantly higher (*p* < 0.05) than those in the controls. Furthermore, the increases in mean PGFM levels in LA-TD (nonmalformed and malformed) were not statistically significant compared with those in V-TD mothers.

### 3.10. Levels of PGEM and PGFM in Maternal Plasma

The mean PGEM plasma levels in the controls were significantly higher (*p* < 0.05) than those in both diabetic mothers. Furthermore, mean PGEM plasma levels in the controls were also significantly higher (*p* < 0.05) than mean PGFM levels in both controls.

There was a substantial increase (*p* < 0.05) in the mean PGFM plasma levels of V-TD and LA-TD compared with controls (Table 11).

The plasma levels of PGEM in LA-TD mothers were significantly higher (*p* < 0.05) than those from V-TD rats. In contrast to the PGEM, the mean PGFM levels in V-TD mothers plasma were significantly higher (*p* < 0.05) than those in LA-TD mothers.

## 4. Discussion

Diabetes during pregnancy is a recognized medical problem. Maternal and embryonic hyperglycemia have been reported to cause congenital malformations in diabetic pregnancy [22]. Congenital anomalies among infants of diabetic mothers represent a fivefold increase over rates observed in the nondiabetic population [23]. The teratogenic effects of diabetes have been largely attributed to various metabolic factors, mainly the increased levels of glucose and ketone bodies [2, 3]. Not that all diabetic embryopathies are prevented by good glycemic control, which indicates that factors other than glucose may participate in the process [24]. Many factors have been proposed as important in the mechanism believed to be causative in diabetic embryopathy, including reduced levels of arachidonic acid [4], alternations in prostaglandin synthesis [2, 4], and increased formation of reactive oxygen species [9, 10].

In the present study, a single injection of streptozotocin in rats resulted in compromised production of insulin, resulting in diabetic rats. This was characterized by elevated glucose levels among these rats as compared to both control groups. Streptozotocin is a potent toxic chemical that damages the beta cells that produce insulin [25].

Endogenous levels of plasma LA are reported to be 1–25 ng/mL in healthy human volunteers [26]. Overall, humans are able to synthesize enough LA to meet their needs for enzyme cofactors. However, its synthesis declines with age and in people with compromised health [27], including diabetes and associated abnormalities, such as diabetic neuropathy. Thus, in these cases, LA may need to be obtained from outside sources by consuming certain foods and from supplements [13]. In this study, the fetal weights from V-TD mothers were significantly less than those in both control and in LA-TD groups, which is in agreement with other studies of diabetic embryopathy* in vivo *[6, 23]. Furthermore, daily injection of LA for 15 days also improved fetal, placental, and membrane mean weight of diabetic rats.

The most widely used technique for measuring gestational development is the fetal CRL, and there is a significant linear relationship between fetal age and CRL [28]. The average CRL of a rat fetus at day 15 of gestation is up to around 14 mm depending on the method used to calculate gestational age [28]. Although the measurements in both groups of our control fetuses were comparable to these figures, the results of this study clearly indicate retardation of fetal development in fetuses of V-TD mothers. The average CRL of fetuses from diabetic mothers was 10.0% shorter than those of the V-TC, and the difference decreased to 4.76% in fetuses from LA-TD mothers as compared to LA-TC. This was indicative of the role of LA in promoting the growth of fetus.

Furthermore, the role of LA in reducing fetal malformations was demonstrated by marginal decrease in the LA-TD rats (42.37%) as opposed to the ones that were not treated (66.66%). Thus, the present study is in agreement with the findings of Sugimura et al. [29], who concluded that LA can reduce neural tube defects (NTDs), cardiovascular malformations, and skeletal malformations in the offspring of diabetic mice at term delivery. In contrast, the preliminary report by Wiznitzer et al. [11] showed that LA failed to exert its protective effects against NTDs in rats treated only during days 1 to 5 of pregnancy but before diabetes induction, which emphasizes the importance of the presence of LA during the organogenesis period (pregnancy days 9 to 11). In the present study, there were no gross fetal morphological malformations exhibited by either control group. This underpins the fact that induced diabetes was the cause of the malformations, reaffirming the findings of Lenhard and Kinsley [30] who reported that diabetes mellitus (DM) is known to cause malformations of fetuses during pregnancy.

Diabetes further weakens the antioxidant system, especially the enzyme for glutathione (GSH) synthesis and gamma-glutamylcysteine ligase (*γ*-GCS), thereby reducing GSH concentration [31]. A previous report by Borcea et al. [32] demonstrated in a cross-sectional study of diabetic patients that those taking LA (600 mg/day for more than 3 months) had decreased oxidative stress compared with those without LA treatment, irrespective of their poor glycemic control and albuminuria. As an antioxidant, LA directly terminates free radicals, chelates transition metal ions, and increases cytosolic glutathione and levels of vitamins C and E [13, 31]. Our results revealed higher total glutathione levels in all LA-treated (control and diabetic) groups compared with nontreated groups and were more pronounced in maternal liver and plasma from LA-TC rats, which is consistent with Kagan et al. [14]. Furthermore, in V-TD rats, the total glutathione levels were reduced in maternal liver (32.24%) and in plasma (70.27%) as compared to V-TC mothers. Thus, the present study reaffirms the findings of Calabrese et al. [33], who reported affiliation of glutathione deficiency with DM.

Although LA supplementation to the diabetic mothers increased total glutathione content in their fetuses as compared to fetuses from V-TD and restored the total glutathione maternal liver to that of the control groups, LA treatment failed to prevent the occurrence of malformations completely. Therefore, it is possible that not all the oxygen-free radicals could be neutralized by LA and that other factors, such as arachidonic acid deficiency and altered prostaglandin metabolism [6, 34], may be involved in the pathogenesis of the diabetes-induced congenital malformations.

A limitation of this study is that we did not measure fetal LA levels after LA supplementation to determine whether maternal diabetes decreased fetal LA levels and if LA supplementation reversed fetal LA levels.

Cyclooxygenase-2 and PGE_2_ have been linked to angiogenesis [35]. It has been suggested that COX-2, but not COX-1, has a crucial role during the initial stages of pregnancy [36]. Our findings that the activity of COX-1 represented only a small percentage of total COX activity are in agreement with data reported previously [6].

In the present study, the specific activity of COX-2 (in malformed fetuses) of V-TD and LA-TD mothers was significantly lower compared with that in fetuses from both the controls and nonmalformed V-TD and LA-TD mothers. Thus, the present study is in line with others' findings [37], which reported inhibition of COX-2 expression (in malformed fetuses) from day 9 to day 11 of gestation.

Furthermore, the activity of COX-2 in membranes from nonmalformed fetuses of LA-TD was significantly higher (*p* < 0.05) than those in nonmalformed fetuses from V-TD rats. This may suggest a protective effect of LA on fetal outcome.

A functional decrease in the amount of prostaglandins may be associated with oxidative stress. Whorton et al. [38] showed that hydrogen peroxide inhibits prostaglandins in cultured endothelial cells. Hempel and Wessels [39] directly demonstrated that oxidative stress decreased PGE_2_ in a dose-dependent manner in a fibroblast system. They also showed that extreme glutathione depletion results in decreased PGE_2_ formation. Glutathione is important in prostaglandin production. Reduced glutathione can serve as a reducing cofactor for the peroxidase component of prostaglandin H synthase (PGSH) system, and it is a required cofactor for PGE isomerase.

Previous studies by Madore et al. [40] and Arosh et al. [41] suggested that the biosynthesis of PGs is favorably directed towards PGE_2_ production rather than PGF_2_ *α* as the conversion of PGH_2_ to PGE_2_ is 150-fold greater than the conversion of PGH_2_ to PGF_2_ *α*. The data from the present study for levels of PGEM and PGFM are not in concordance with those reports. The results from this study and another study [6] showed that, in V-TC fetuses, the levels of PGEM and PGFM were nearly equal while, in the placentas and membranes, the level of PGEM was higher than that of PGFM. The general trend was that the levels of PGEM were lower in both the diabetic groups—mainly in those associated with malformation—and had lower total glutathione contents. On the other hand, PGFM levels were higher in the malformed diabetic fetuses, followed by diabetic fetuses that were LA-treated.

Furthermore, the fetuses, placentas, and membranes from LA-TD mothers demonstrated relatively higher PGEM levels. This further demonstrates that LA plays a very important role in trying to maintain PGEM and PGFM levels within normal levels in the body.

The elevated levels of PGFM in the malformed fetuses of both diabetic groups reported in the present study may reflect oxidative stress status, as has been suggested by El-Bassiouni et al. [42]. Yin et al. [43] suggested that PGF_2_ *α* in humans is derived from the isoprostane pathways, which are PG-like compounds formed* in vivo* from the free radical-catalysed peroxidation of arachidonic acid without the activity of COX enzyme, an alternative route for the formation of PGF_2_ *α in vivo*.

Prostaglandin metabolism in rats follows the same route as in humans and is different from those in mice and rabbits [44]. Considering that the findings from the present study are comparable to those in humans would, therefore, be justified. The formation through this pathway is noticeably increased in cases of oxidative stress and other inflammatory conditions [43, 45]. Thus, this could explain the elevated levels and increased production of PGF_2_ *α* in diabetic gestation. This dogma has further support from the Stephens et al. [46] study, which reported that diabetic patients have elevated serum levels of thiobarbituric acid reactive substances F2 isoprostanes and 8-OH-guanosine compared with nondiabetics.

## 5. Conclusions

This research therefore determines that administration of LA to diabetic pregnant mothers can protect against weight loss and promote the fetal growth developments. Furthermore, diabetes and thus malformation are associated with lower total glutathione contents. LA supplementation to diabetic mothers restored maternal liver total glutathione content to that of control groups.

LA treatment to diabetic mothers failed to normalize activities of COX-2 in fetuses and placentas to those in control rats and increased levels of PGEM in malformed fetuses as compared to V-TD but failed to normalize it to levels of both groups of control rats. In contrast to PGEM, the greatest increases in PGFM were found in malformed fetuses from V-TD and LA-TD mothers. Furthermore, LA supplementation increased plasma levels of PGEM than those found in V-TD rats.

Since LA treatment did not completely prevent the occurrence of malformations, other factors may be involved in the pathogenesis of diabetes-induced congenital malformations. Hence, the possibility of combining LA with other effective agents such as vitamin E or folic acid to prevent malformations in fetuses of mothers with diabetes is interesting.

## Figures and Tables

**Table 1:** Morphological outcome, resorption rate, and blood glucose among the control and the diabetic groups.

Groups	Number of litters	Maternal body weight (g)	Body weight gain (g)	Number of viable fetuses	Mean implantation/litter	Number of resorption processes	Blood glucose Mmol/L
Vehicle-treated control (V-TC)	9	211.30 ± 1.35	38.77 ± 2.05	69	7.66 ± 0.62	0.00	4.88 ± 0.55
LA-treated control (LA-TC)	7	213.74 ± 1.69	39.27 ± 5.42	61	8.71 ± 0.75	0.00	5.49 ± 0.83
Vehicle-treated diabetic (V-TD)	10	208.23 ± 1.44	5.67 ± 1.60^†^	48	6.00 ± 1.07	5.80 ± 2.15^*∗∗*^	25.42 ± 1.54^*∗*^
LA-treated diabetic LA-TD)	8	209.26 ± 1.57	10.12 ± 2.37	59	7.37 ± 0.73	0.00	21.92 ± 2.21^*∗*^

^*∗*^Significantly higher (*p* < 0.05) than those from both groups of control rats.

^*∗∗*^Significantly higher (*p* < 0.03) than those from both groups of control and LA-TD rats.

^†^Significantly lower (*p* < 0.05) than those from both groups of control and LA-TD rats.

**Table 2:** The fetal body weight, placental weight, the membrane weight, and the crown-rump length.

Groups	Fetal body weight (mg)	Placental weight (mg)	Membrane weight (mg)	Crown-rump length (CRL) (mm)
Vehicle-treated control (V-TC) (*n* = 69)	490.23 ± 14.13	275.78 ± 9.68	93.94 ± 5.98^†^	15.55 ± 0.16
LA-treated control (LA-TC) (*n* = 61)	504.52 ± 29.47	300.59 ± 9.65	96.47 ± 4.35^†^	15.73 ± 0.32
Vehicle-treated diabetic (V-TD) (*n* = 48)	347.55 ± 17.93^*∗*^	305.77 ± 9.35^*∗∗*^	68.04 ± 4.07	13.99 ± 0.24^*∗*^
LA-treated diabetic (LA-TD) (*n* = 59)	468.52 ± 42.46	338.03 ± 18.57^*∗∗*^	83.10 ± 4.15	14.98 ± 0.45

^*∗*^Significantly lower (*p* < 0.05) than those from both groups of control and LA-TD rats.

^*∗∗*^Significantly higher (*p* < 0.05) than those from V-TC rats.

^†^Significantly higher (*p* < 0.05) than those from both groups of diabetic rats.

The difference in the mean values of the two groups is statistically significant difference using Shapiro-Wilk, Kruskal-Wallis, and Tukey test one way analysis of variance. The data represent mean ± SEM.

**Table 3:** Malformations present in the two diabetic groups (vehicle- and LA-treated group).

	Vehicle-treated diabetic (V-TD)		LA-treated diabetic (LA-TD)	
	Nonmalformed	Malformed	Nonmalformed	Malformed
Fetal number	16	32 (66.66%)	34	25 (42.37%)
Fetal body weight (mg)	361.87 ± 22.51	317.00 ± 25.59	568.39 ± 62.12^*∗*†^	357.95 ± 50.41
Placental weight (mg)	317.00 ± 11.25	281.81 ± 15.58	357.14 ± 24.76^††^	316.87 ± 27.85
Membrane weight (mg)	71.34 ± 5.13	61.00 ± 6.41	95.05 ± 7.15^*∗*†^	69.87 ± 6.69
CRL (mm)	14.03 ± 0.31	13.92 ± 0.36	15.57 ± 0.62^†^	14.44 ± 0.60
Short mandible		19 (39.58%)		6 (10.17%)
Open neural tube		15 (31.25%)		8 (13.56%)
Malrotation		6 (12.5%)		3 (5.09%)
Short tail		1 (2.08%)		0 (0.00%)

^*∗*^Significantly higher (*p* < 0.05) compared with malformed LA-TD.

^†^Significantly higher (*p* < 0.05) compared with V-TD rats (nonmalformed and malformed).

^††^Tend to increase more than malformed V-TD (*p* = 0.06).

There are no significant relationships between placental and membrane body weights of any groups.

The difference in the mean values of the two groups is statistically significantly different using Shapiro-Wilk and Mann-Whitney one way analysis of variance rank sum test.

**Table 4:** Total glutathione content in maternal liver and plasma and in fetuses of both control and diabetic groups.

Groups	Maternal liver (*μ*mol/g)	Plasma (*μ*mol)	Fetuses (*μ*mol/mg of protein)
Vehicle-treated control (V-TC)	4.28 ± 0.61 (*n* = 9)	1.85 ± 0.21	8.85 ± 0.32 (*n* = 69)
LA-treated control (LA-TC)	4.98 ± 0.72 (*n* = 7)	2.12 ± 0.31	9.43 ± 0.93 (*n* = 61)
Vehicle-treated diabetic (V-TD)	2.90 ± 0.31^*∗*^ (*n* = 10)	0.55 ± 0.31^*∗*^	4.32 ± 0.82^*∗*†^ (*n* = 48)
LA-treated diabetic (LA-TD)	3.91 ± 0.40 (*n* = 8)	1.51 ± 0.12	6.17 ± 0.32^*∗∗*†^ (*n* = 59)

^*∗*^Significantly lower (*p* < 0.05) compared with both groups of control and LA-TD rats.

^*∗∗*^Significantly increased (*p* < 0.05) compared with those in V-TD rats.

^†^Significantly lower (*p* < 0.05) compared with both groups of control rats.

**Table 5:** COX activity in fetuses (U/mg of protein).

Groups	Total COX		COX-1		COX-2	
Vehicle-treated control (V-TC) (*n* = 69)	91.21 ± 5.93		12.24 ± 2.20		78.96 ± 6.52	
LA-treated control (LA-TC) (*n* = 61)	94.30 ± 4.22		14.33 ± 2.45		79.97 ± 7.89	
	Nonmalformed	Malformed^*∗*^	Nonmalformed	Malformed	Nonmalformed	Malformed^*∗*^
Vehicle-treated diabetic (V-TD) (*n* = 48)	88.74 ± 4.16	52.80 ± 2.11	11.31 ± 1.24	10.28 ± 1.43	77.43 ± 5.28	42.52 ± 2.54
LA-treated diabetic (LA-TD) (*n* = 59)	86.89 ± 6.46	60.54 ± 4.02	12.47 ± 6.20	8.58 ± 1.34	74.43 ± 5.83	47.96 ± 3.04

^*∗*^Significantly lower (*p* < 0.05) compared with fetuses from V-TC, LA-TC, and nonmalformed groups of both groups of diabetic rats.

**Table 6:** COX activity in placentas (U/mg of protein).

Groups	Total COX		COX-1		COX-2	
Vehicle-treated control (V-TC) (*n* = 69)	96.72 ± 7.96		8.59 ± 0.75		88.13 ± 6.39^*∗∗*^	
LA-treated control (LA-TC) (*n* = 61)	86.42 ± 6.87		9.24 ± 0.81		77.18 ± 5.45^*∗∗*^	
	Nonmalformed	Malformed^*∗*^	Nonmalformed	Malformed^*∗*^	Nonmalformed	Malformed^*∗*^
Vehicle-treated diabetic (V-TD) (*n* = 48)	53.65 ± 7.28	33.60 ± 4.04	9.21 ± 1.01	6.62 ± 0.47	44.44 ± 5.02	26.98 ± 2.71
LA-treated diabetic (LA-TD) (*n* = 59)	63.59 ± 6.72	43.51 ± 5.08	10.04 ± 1.10	7.03 ± 0.50	53.55 ± 6.05	36.43 ± 3.66^†^

^*∗*^Significantly lower (*p* < 0.05) compared with placentas of nonmalformed fetuses of both groups of diabetic rats.

^*∗∗*^Significantly higher (*p* < 0.05) compared with placentas of both nonmalformed and malformed fetuses of diabetic rats.

^†^Significantly higher (*p* < 0.05) than those in malformed fetuses from V-TD rats.

**Table 7:** COX activity membranes (U/mg of protein).

Groups	Total COX		COX-1		COX-2	
Vehicle-treated control (V-TC) (*n* = 9)	115.53 ± 7.51		8.64 ± 0.23		106.89 ± 6.81	
LA-treated control (LA-TC)) (*n* = 7)	127.05 ± 9.84		10.15 ± 1.39		116.90 ± 8.70	
	Nonmalformed^†^	Malformed^†^	Nonmalformed	Malformed	Non-malformed^††^	Malformed^*∗∗*^
Vehicle-treated diabetic (V-TD) (*n* = 10)	80.54 ± 6.40	60.40 ± 4.22	10.07 ± 1.46	9.07 ± 0.78	70.47 ± 3.53	51.33 ± 4.18
LA-treated diabetic (LA-TD) (*n* = 8)	92.75 ± 7.42^*∗*^	65.82 ± 5.46	8.98 ± 0.92	8.73 ± 0.41	83.77 ± 5.02^*∗∗∗*^	57.09 ± 6.56

^†^Significantly lower (*p* < 0.05) in comparison to membranes from both control fetuses.

^††^Significantly lower (*p* < 0.05) than those in the both control fetuses.

^*∗*^Significantly higher (*p* < 0.05) compared with the nonmalformed fetuses of V-TD rats.

^*∗∗*^Significantly lower (*p* < 0.05) than those in the membranes of fetuses from V-TC, LA-TC, and nonmalformed fetuses from both groups of diabetic rats.

^*∗∗∗*^Significantly higher (*p* < 0.05) than those in nonmalformed fetuses from V-TD rats.

**Table 8:** PGEM and PGFM in fetuses.

Groups	PGEM (pg/mg of protein)		PGFM (pg/mg of protein)	
	Fetuses			
Vehicle-treated control (V-TC) (*n* = 69)	133.11 ± 13.56		132.56 ± 12.51	
LA-treated control (LA-TC) (*n* = 61)	113.51 ± 8.28		120.06 ± 8.92	
	Nonmalformed^†*∗*^	Malformed^†^	Nonmalformed	Malformed
Vehicle-treated diabetic (V-TD) (*n* = 48)	92.64 ± 6.95	67.33 ± 2.71	109.24 ± 10.84	159.54 ± 8.97^*∗∗∗*^
LA-treated diabetic (LA-TD) (*n* = 59)	109.35 ± 8.38	75.23 ± 3.32^*∗∗*^	115.14 ± 7.05	155.80 ± 9.40^*∗∗∗*^

^†^Significantly lower (*p* < 0.05) than those in both controls.

^*∗*^Significantly higher (*p* < 0.05) than those in the malformed fetuses of both groups of diabetic rats.

^*∗∗*^Significantly higher (*p* < 0.05) than those in malformed fetuses from V-TD rats.

^*∗∗∗*^Significantly higher (*p* < 0.05) than those in nonmalformed fetuses of V-TD, LA-TD, and both groups of control rats.

**Table 9:** PGEM and PGFM in placentas.

Groups	PGEM (pg/mg of protein)		PGFM (pg/mg of protein)	
	Placentas			
Vehicle-treated control (V-TC) (*n* = 69)	120.57 ± 7.84		76.17 ± 4.31	
LA-treated control (LA-TC) (*n* = 61)	110.50 ± 5.98		81.96 ± 5.91	
	Nonmalformed^*∗*^	Malformed^*∗*†^	Nonmalformed^*∗∗*^	Nonmalformed^*∗∗∗*^
Vehicle-treated diabetic (V-TD) (*n* = 48)	85.93 ± 10.48	40.11 ± 3.80	98.26 ± 7.29	120.45 ± 11.02
LA-treated diabetic (LA-TD) (*n* = 59)	74.46 ± 11.38	45.41 ± 3.85	108.51 ± 8.85	123.34 ± 9.46

^†^Significantly lower (*p* < 0.05) than those in the control groups and in nonmalformed fetuses of both groups of diabetic rats.

^*∗*^Significantly lower (*p* < 0.05) than placentas from both groups of control fetuses.

^*∗∗*^Significantly higher (*p* < 0.05) than those in both groups of control fetuses.

^*∗∗∗*^Significantly higher (*p* < 0.05) than those from nonmalformed fetuses of both diabetic and control groups.

**Table 10:** PGEM and PGFM in membranes.

Groups	PGEM (pg/mg of protein)		PGFM (pg/mg of protein)	
	Membranes			
Vehicle-treated control (V-TC) (*n* = 9)	96.95 ± 6.56		56.50 ± 4.51	
LA-treated control (LA-TC) (*n* = 7)	85.99 ± 5.69		65.69 ± 5.23	
	Nonmalformed^*∗*^	Malformed^*∗*†^	Nonmalformed^††^	Malformed^††^
Vehicle-treated diabetic (V-TD) (*n* = 10)	55.32 ± 4.67	28.09 ± 1.83	82.13 ± 4.68	88.30 ± 4.94
LA-treated diabetic (LA-TD) (*n* = 8)	69.02 ± 4.48^*∗∗*^	35.77 ± 2.41^*∗∗*^	90.38 ± 6.85	96.89 ± 6.49

^*∗*^Significantly lower (*p* < 0.05) than those in the controls.

^*∗∗*^Significantly increased (*p* < 0.05) compared with those in the V-TD fetuses.

^†^Significantly below (*p* < 0.05) those of nonmalformed fetuses of both diabetic groups.

^††^Significantly higher (*p* < 0.05) than those in control groups.

**Table 11:** PGEM and PGFM in maternal plasma.

Groups	Maternal plasma (pg/mg of protein)	
	PGEM	PGFM
Vehicle-treated control (V-TC) (*n* = 9)	73.13 ± 3.78^*∗*^	58.72 ± 2.48
LA-treated control (LA-TC) (*n* = 7)	79.90 ± 4.56^*∗*^	63.10 ± 2.50
Vehicle-treated diabetic (V-TD) (*n* = 10)	51.89 ± 0.96	88.92 ± 1.48^†*∗∗∗*^
LA-treated diabetic (LA-TD) (*n* = 8)	56.81 ± 1.45^*∗∗*^	84.18 ± 1.32^†^

^*∗*^Significantly higher (*p* < 0.05) than those in both diabetic mothers and higher than PGFM levels in both controls.

^*∗∗*^Significantly higher (*p* < 0.05) than those from V-TD rats.

^*∗∗∗*^Significantly higher (*p* < 0.05) than those in LA-TD rats.

^†^Significantly higher (*p* < 0.05) compared with controls.

## Abbreviations

COX: Cyclooxygenase
DM: Diabetes mellitus
LA: Lipoic acid
PG: Prostaglandin
PGEM: Prostaglandin E_2_ metabolite
PGFM: Prostaglandin F_2_ *α* metabolite
V-TC: Vehicle-treated control
LA-TC: LA-treated control
V-TD: Vehicle-treated diabetic
LA-TD: LA-treated diabetic.

## Additional Points

*Availability of Data and Materials.* The datasets supporting the conclusions of this article are included within the article.
