# Corrigendum to “Effects of Lipoic Acid Supplementation on Activities of Cyclooxygenases and Levels of Prostaglandins E_2_ and F_2_*α* Metabolites, in the Offspring of Rats with Streptozotocin-Induced Diabetes”

**DOI:** 10.1155/2017/8135610

**Published:** 2017-07-12

**Authors:** Hisham Y. Al-Matubsi, Ghaleb A. Oriquat, Mahmoud Abu-Samak, Othman A. Al Hanbali, Maher D. Salim

**Affiliations:** ^1^Faculty of Pharmacy, University of Petra, Amman, Jordan; ^2^Faculty of Pharmacy and Medical Sciences, Al-Ahliyya Amman University, Amman, Jordan; ^3^Faculty of Pharmacy, Applied Science Private University, Amman, Jordan; ^4^Faculty of Pharmacy, Al-Zaytoonah University, Amman, Jordan; ^5^Middle East University, Amman, Jordan

In the article titled “Effects of Lipoic Acid Supplementation on Activities of Cyclooxygenases and Levels of Prostaglandins E_2_ and F_2_*α* Metabolites, in the Offspring of Rats with Streptozotocin-Induced Diabetes” [1], affiliation number three was incorrect. The correct affiliation list is shown above. Additionally, there was an error in the Acknowledgments section, which should be corrected as follows:

“This work was supported by grants from Applied Science Private University (REP-LA 2010/H) and Al-Ahliyya Amman University (LAG20102106/g). The funders had no role in the design of the study, data collection, analysis, and interpretation of data or in writing the manuscript.”
